# Spoilage Potential of Contaminating Yeast Species *Kluyveromyces marxianus*, *Pichia kudriavzevii* and *Torulaspora delbrueckii* during Cold Storage of Skyr

**DOI:** 10.3390/foods11121776

**Published:** 2022-06-16

**Authors:** Thanyaporn Srimahaeak, Mikael Agerlin Petersen, Søren K. Lillevang, Lene Jespersen, Nadja Larsen

**Affiliations:** 1 Department of Food Science, University of Copenhagen, Rolighedsvej 26, 1958 Frederiksberg, Denmark; thanya@food.ku.dk (T.S.); map@food.ku.dk (M.A.P.); lj@food.ku.dk (L.J.); 2 Arla Foods Innovation Centre, Agro Food Park 19, 8200 Aarhus, Denmark; sklv@arlafoods.com

**Keywords:** yeasts, *Kluyveromyces marxianus*, skyr, volatile compounds, spoilage

## Abstract

This study investigated the spoilage potential of yeast strains *Kluyveromyces marxianus* (Km1, Km2 and Km3)*, Pichia kudriavzevii* Pk1 and *Torulaspora delbrueckii* Td1 grown in skyr in cold storage. Yeast strains were isolated from skyr and identified by sequencing of the 26S rRNA gene. *K. marxianus* yeasts were grown in skyr to high numbers, generating large amounts of volatile organic compounds (VOC) associated with off-flavours, among them were alcohols (3-methyl-1-butanol, 2-methyl-1-propanol and 1-hexanol), esters (ethyl acetate and 3-methylbutyl acetate) and aldehydes (hexanal, methylbutanal and methylpropanal). Growth of *P. kudriavzevii* Pk1 led to moderate increases in several alcohols and esters (mostly, 3-methyl-1-butanol and ethyl acetate), whereas only minor shifts in VOCs were associated with *T. delbrueckii* Td2. The levels of the key aroma compounds, diacetyl and acetoin, were significantly decreased by all *K. marxianus* strains and *P. kudriavzevii* Pk1. In contrast to the other yeast species, *K. marxianus* was able to utilize lactose, producing ethanol and carbon dioxide. Based on the overall results, *K. marxianus* was characterised by the highest spoilage potential. The study revealed the differences between the yeast species in fermentative and spoilage activities, and clarified the role of yeast metabolites for off-flavour formation and quality defects in skyr during cold storage.

## 1. Introduction

Skyr is a traditional Icelandic dairy product, which nowadays has become popular in many countries of Europe and North America. In the UK and Denmark, the current share of skyr in the total yoghurt market is as high as 24% [1]. Skyr is characterised by its high protein content (10–15%), creamy smooth texture and typical sour dairy flavour [2]. Though skyr is classified in the market as a yoghurt-type product, it is technically a fresh cheese, traditionally made with addition of rennet, an enzyme used for milk coagulation and curd formation [2,3]. Today, most of the modernized dairies solely use starter cultures instead of rennet in skyr production. In contrast to yoghurt, which is typically made from full fat milk, skyr is produced from skimmed milk, and whey is removed after milk fermentation by either centrifugation or ultrafiltration [3]. The major starter cultures in skyr and yoghurt comprise strains of *Streptococcus thermophilus* and *Lactobacillus delbrueckii* subsp. *bulgaricus*. The role of starter cultures for yoghurt quality has been widely studied, while for skyr this knowledge is insufficient. In yoghurt, a desired aroma depends on the balanced amounts of organic acids and the key aroma compounds such as acetaldehyde, diacetyl, acetoin, acetone and 2-butanone [4,5]. Tian and co-workers reported that the appealing flavour of yoghurt depended on the appropriate concentration of diacetyl, acetaldehyde and acetoin, with the optimal ratio of 4:16:32 mg/L, respectively [6]. Other VOCs, including aldehydes and ketones produced by lipid oxidation, as well as fatty acids and the products of the Maillard reaction (e.g., furan derivatives), have been associated with off-flavours in yoghurts [5,6,7].

In former times, the yeasts *Candida famata* (teleomorph *Debaryomyces hansenii*, formally *Torulopsis candida*), *Zygosaccharomyces bailii* (formerly *Saccharomyces bailii*), *Saccharomyces delbrueckii* var. *mongolicus* (syn. *Torulaspora delbrueckii*) and *Saccharomyces cerevisiae* were used in skyr manufacture to create a product with a unique texture and flavour, lower acidity and a tiny amount of alcohol [3]. In the modern dairy industry, yeasts are rather considered as contaminants and potential spoilage microorganisms. The common yeast spoilers in yoghurt and skyr belong to the genera *Kluyveromyces*, *Debaryomyces*, *Pichia*, *Candida* and *Saccharomyces*. Whether a given yeast species can be regarded as a contaminant depends on its ability to propagate at refrigerated temperatures, acidic pH and low water activity during the storage of the dairy products [8,9]. When grown in high numbers (typically more than 10^5^ CFU/g), yeasts can cause quality deterioration and shorten the product’s shelf life. The most common quality defects, resulting from the metabolic activity of yeasts, include the production of gas, off-flavours, discolouration and changes in texture [10,11].

*Kluyveromyces marxianus* is well known for its biotechnological applications, such as the production of various enzymes, bioethanol, phenylethanol and fructose [12]. Due to the respiratory-fermentative type of metabolism, similar to *Saccharomyces cerevisiae*, *K. marxianus* has been successfully used as a baker’s yeast for the improvement of bread sensorial qualities [13,14,15]. In yoghurt-type products, *K. marxianus* is considered as a major spoilage yeast [10,11,12,13,14,15,16]. Though *K. marxianus* is designated as GRAS (generally recognized as safe) and rarely associated with health concerns, the quality and preservation of the dairy products contaminated with this species would still be of concern. It is well known that *K. marxianus* has the ability to metabolize lactose, citrate, protein and fat in milk, and produce a variety of secondary metabolites (e.g., acetaldehydes, carbon dioxide and ethanol), which might impart aroma development in dairy products [17,18]. Total sensory evaluation revealed an extensive negative impact of *K. marxianus* on the sensory qualities of yoghurts, which was even greater than the non-lactose-fermenting yeast *D. hansenii* [19]. Fermentation of residual lactose within the yoghurt curd by *K. marxianus* and the release of carbon dioxide formed cracks, loosening the curd texture [19]. Zhang and co-workers reported that *K. marxianus* promoted proteolysis in fermented milk, generating high amounts of free amino acids and small peptides [20]. Other yeast species used in this study, *P. kudriavzevii* (formerly *Issatchenkia orientalis* and anamorph *Candida krusei*) and *Torulaspora delbrueckii*, have been described as non-lactose-fermenting yeasts, sporadically found in kefir grains, yoghurts, cheeses and African fermented foods [21,22,23]. Due to a strong proteolytic activity, *P. kudriavzevii* can be part of the ripening cultures in some cheeses (e.g., Kazak artisanal cheese in China and Armanda cheese in Spain), producing a range of desired aroma compounds [24]. In other dairy products, specifically at a low pH, *P. kudriavzevii* might form surface biofilms and present a major cause of fermentative spoilage [17].

Most of the published studies have been primarily focused on yeast biocontrol, growth characteristics and identification in dairy products. Only a few studies with fermented milks, particularly cheeses, examined the production of flavour compounds by *K. marxianus*. To our knowledge, data on the impact of yeasts on the quality and aroma development in skyr during storage have not been published so far. The aim of the present study was to evaluate the spoilage potential of the yeasts *K. marxianus*, *P. kudriavzevii* and *T. delbrueckii*, grown in skyr at a cold storage temperature, with a focus on the production of volatile aroma compounds, organic acids and other metabolites related to off-flavours and other quality defects.

## 2. Materials and Methods

### 2.1. Yeast Strains and Reagents

The strains of *Kluyveromyces marxianus* Km1, Km2 and Km3 were isolated from contaminated batches of skyr provided by a Danish dairy. Before isolation, skyr was incubated at 25 °C for 2 weeks to promote the growth of yeast contaminants. Afterwards, 20 g of skyr was mixed with 180 mL peptone–saline solution (SPO; 5.0 g NaCl; 0.3 g Na_2_HPO_4_·2H_2_O; 1.0 g Bacto Peptone per litre of distilled water; pH 5.6), homogenized in a stomacher (Struers Chem A/S, Hovedstaden, Denmark) for 120 s at high speed and plated on malt yeast glucose peptone agar (MYGP; 3.0 g malt extract; 3.0 g yeast extract; 10 g glucose; 5.0 g Bacto peptone; 15 g agar, pH 5.6). Then, 100 mg·L^−1^ chloramphenicol and 50 mg·L^−1^ chlortetracycline was added to inhibit bacterial growth. MYGP agar plates were incubated at 25 °C for 48 h. Yeast strains *Pichia kudriavzevii* Pk1 and *Torulaspora delbrueckii* Td1 were previously isolated from skyr production and provided by Arla Foods (Copenhagen, Denmark). Yeast isolates were stored at −80 °C in MYGP broth containing 20% (*w*/*v*) glycerol. All reagents for the experiments were purchased from Sigma-Aldrich (Copenhagen, Denmark) or Thermo Fisher Scientific (Copenhagen, Denmark) unless otherwise stated.

### 2.2. Sequencing of 26S rRNA Gene and 5.8S-ITS Region

Total yeast DNA was extracted from the colony material using an InstaGene Matrix DNA extraction kit (Bio-Rad, Hercules, CA, USA). The PCR was performed in a 50 μL mixture, containing 25 μL of Taq DNA Polymerase 2× Master Mix RED (Ampliqon, Odense, Denmark), 5 μL primer mix and 3 μL of the sample DNA. The D1/D2 region of the 26S rRNA gene was amplified, using the primers NL-1 (5′-GCATATCAATAAGCGGAGGAAAAG-3′) and NL-4 (5′-GGTCCGTGTTTCAAGACGG-3′) [25]. The PCR temperature cycling conditions included initial denaturation at 95 °C for 5 min, 35 cycles of 95 °C for 1 min, annealing at 52 °C for 45 s and extension at 72 °C for 1 min, followed by final extension at 72 °C for 7 min. Additionally, sequencing of the internal transcribed spacer region (ITS1-5.8S rRNA-ITS2) was applied to differentiate the species of *K. marxianus* and *K. lactis*. Primers used for amplification of the 5.8S-ITS region were ITS-1 (5′-TCC GTA GGT GAA CCT GCG G-3′) and ITS-4 (5′- TCC TCC GCT TAT TGA TAT GC-3′) [26]. The PCR conditions included initial denaturation at 95 °C for 3 min, 35 cycles of 95 °C for 30 s, 60 °C for 45 s and 72 °C for 45 s, followed by final extension at 72 °C for 10 min. The PCR products were sequenced by Macrogen Europe (Amsterdam, The Netherlands).

To identify yeast isolates, sequences were aligned with 26S rRNA gene and ITS1-5.8S rRNA-ITS2 region sequences in the NCBI Genbank database, using the basic local alignment search tool (BLAST) (http://www.ncbi.nlm.nih.gov/BLAST/, accessed on 20 September 2021).

### 2.3. Pulsed-Field Gel Electrophoresis

Pulsed-field gel electrophoresis (PFGE) was performed to characterise the chromosome length polymorphism among the dairy yeast isolates, compared to the type strains of *K. marxianus* CBS1553 and *K. lactis* CBS845 [27]. In brief, yeast chromosomal DNA was loaded into agarose plugs using the CHEF Yeast Genomic DNA plug kit (BIO-RAD, Coppenhagen, Denmark). PFGE was carried out on a PFGE DR-III unit (BIO-RAD, Hercules, CA, USA) at 10 °C in TBE buffer (45 mM Tris-base, 44 mM boric acid and1 mM EDTA). The running conditions were 150 V at a 200 s switch interval for 24 h, followed by 100 V at a 700 s switch interval for 48 h. *Saccharomyces cerevisiae* 345S (BIO-RAD, Hercules, CA, USA) and *Hansenula wingei* (BIO-RAD, Hercules, CA, USA) were used as chromosomal markers. The gel was stained in ethidium bromide and visualized with UV transillumination (Alpha-InnoTec GmbH, Kasendorf, Germany). BioNumerics software (version 2.50, Applied Maths, Kortrijk, Belgium) was used for cluster analysis of the DNA profiles.

### 2.4. Yeast Propagation in Skyr

Skyr (natural skyr, 0.2% fat and 11% protein) was received from a Danish dairy. Skyr was produced with the use of the starter cultures *Lactobacillus delbrueckii* subsp. *bulgaricus* and *Streptococcus thermophilus*, and a bioprotective culture *Lacticaseibacillus paracasei.* Before the experiments, yeasts were propagated in MYGP broth at 25 °C overnight, washed and resuspended in SPO. Skyr was aseptically distributed in stomacher bags (200 g in each) and inoculated with yeast cell suspensions (2 mL) to a final concentration of 10^4^ CFU·g^−1^ or with SPO (2 mL) for the control experiments. The mixtures were homogenized in a stomacher (Struers Chem A/S, Coppenhagen, Denmark) for 120 s at high speed, distributed into 50 mL sterile polypropylene boxes (Berry Superfoss 618 PP plast, Dansk Transport Emballage A/S, Vojens, Denmark) and incubated at 8 °C. Samples were withdrawn at the start (Day 0) and after 7, 14 and 21 days of incubation. Samples were stored at −20 °C before the analyses.

### 2.5. Measurements of Viable Counts, pH and Carbon Dioxide

Viable counts of yeasts in skyr were determined by plating serial dilutions in SPO onto MYGP agar plates with added antibiotics (100 mg·L^−1^ chloramphenicol and 50 mg·L^−1^ chlortetracycline), which were incubated at 25 °C for 48 h. Lactic acid bacteria (LAB) were enumerated using de Man, Rogosa Sharpe (MRS, pH 6.2), containing an antifungal agent (0.01% cycloheximide). The MRS agar plates were incubated anaerobically using AnaeroGen sachet (Thermo Fisher Scientific, Roskilde, Denmark) at 37 °C for 48 h. The pH in skyr samples was recorded using a digital pH meter (Mettler Toledo, Glostrup, Denmark). Production of CO_2_ was calculated from the total weight loss of skyr during incubation and expressed in nmol·g^−1^ of CO_2_ [28].

### 2.6. GC-MS Analysis of the Volatile Compounds and Data Processing

Volatile compounds in skyr were analysed using headspace gas chromatography coupled to mass spectrometry (GC-MS), as previously described [29]. Briefly, a mixture of 30 g of skyr, 30 mL of tap water and 1 mL of internal standard (4-methyl-1-pentanol, 5 mg·L^−1^) was homogenized using an Ultra-Turrax T25 homogenizer (Janke & Kunkel, Staufen, Germany) at 12,000 rpm for 90 s. VOCs were collected onto Tenax-TA traps (Markes International, Llantrisant, UK) by purging with nitrogen (100 mL·min^−1^) for 45 min and a dry purge of the traps for 10 min. The volatiles were desorbed by an automatic thermal desorption unit (TurboMatrix 350, Perkin Elmer, Shelton, CT, USA) into a GC-MS (7890A GC-system interfaced with a 5975C VL MSD with triple-Axis detector, Agilent Technologies, Palo Alto, California) and separated on a DB-Wax capillary column. The mass spectra were recorded at 70 eV within a mass/charge ratio of 15–300 m/z. Data were extracted from the chromatograms using the PARADISe software [30]. Volatile compounds were identified from the retention indices (RI), and calculated using a series of n-alkanes (C_6_–C_22_) and the NIST05 database. Identifications were confirmed by comparison with the RI of authentic reference compounds or published RI values. Semi-quantitative data for volatiles were obtained by multiplying the internal standard concentration with the ratio of the peak area of the volatile compound to the internal standard peak area [31].

### 2.7. High Performance Liquid Chromatography

Organic acids (lactic, citric, acetic, orotic and uric acids), carbohydrates (lactose, glucose and galactose) and ethanol were analysed using high performance liquid chromatography (HPLC) [32,33]. Briefly, 5 g of the skyr test samples and controls were diluted with 0.01 M H_2_SO_4_ to 25 mL and centrifuged at 4000× *g*, 20 min at 4 °C (Eppendorf, Hamburg, Germany). The supernatant was collected by filtering through a 0.2 μm membrane (Sarstedt, Germany) and stored at −20 °C before the analysis. The chromatographic separation was conducted with 10 mM H_2_SO_4_ as a mobile phase at a flow rate of 0.6 mL·min^−1^ on an Aminex HPX-87H column (300 mm × 7.8 mm). The HPLC equipment consisted of a quaternary pump, an online degasser, a UV-visible detector and a refractive index detector (RID; Agilent 1100 series, Waldbronn, Germany). The UV detector was set at 210 nm (citric, lactic and acetic acids) and 290 nm (orotic and uric acid) for the detection of organic acids. The RID detector was used for the analyses of carbohydrates. Data were collected and processed using Agilent ChemStation software (Agilent Technologies, Waldbronn, Germany). Quantification of organic acids was carried out using external standards of the relevant compounds. The calibration curves were constructed for the individual organic acids by plotting the peak heights after duplicate injections of standard solutions, at a broad range of concentrations. Identifications were made from the retention times of the individual standards.

### 2.8. Phenotypic Tests

Yeasts were tested for the ability to ferment and assimilate glucose, lactose and galactose [34]. Thereafter, yeast colony material was resuspended in the media for fermentation tests (0.45% *w*/*v* yeast extract, 0.75% *w*/*v* peptone, 2.66 mg·100 mL^−1^ bromothymol blue, 2% *w*/*v* carbohydrate added, pH 6.0 ± 0.2) and for assimilation tests (0.067% *w*/*v* Difco Yeast Nitrogen Base, 0.05% *w*/*v* carbohydrate added, pH 5.6 ± 0.1). Durham tubes were used to detect gas production in fermentation tests. Gas production and colour change of the indicator (bromothymol blue) was recorded weekly. Yeast growth was scored based on the turbidity of the cell suspensions using Wickerham cards.

### 2.9. Statistical Analysis

All experiments were conducted in triplicate. Significant differences between the results (confidence level of 95%) were tested using one-way analysis of variance (ANOVA) followed by a Tukey’s post-hoc test (JMP software, SAS Institute Inc., Cary, NC, USA) and the unpaired *t*-test (GraphPad Prism 9, GraphPad Software, San Diego, CA, USA).

### 2.10. Nucleotide Sequence Accession Number

The sequences of the D1/D2 region of the 26S rRNA gene were deposited in the DDBJ, EMBL and GenBank nucleotide sequence databases under accession numbers OM854769-OM854773 (Table 1). Sequences of the ITS1-5.8S rRNA gene-ITS2 fragments for *K. marxianus* Km1, Km2 and Km3 were deposited under accession numbers OM406350, OM406351 and OM406352, respectively.

## 3. Results

### 3.1. Yeasts Identification and Growth

**Figure 1:**
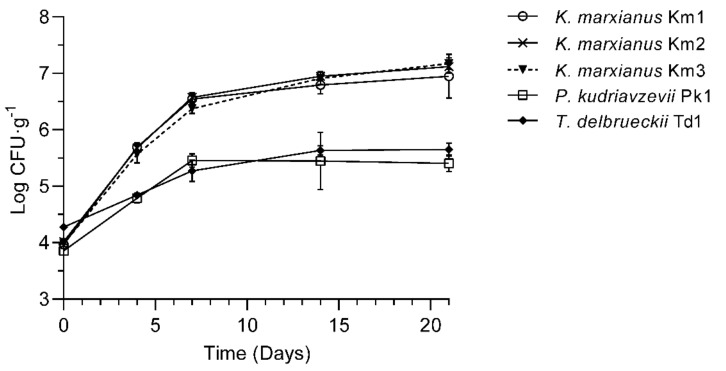
Viable counts (log CFU·g^−1^) of yeasts *K. marxianus* (strains Km1, Km2 and Km3), *P. kudriavzevii* Pk1 and *T. delbrueckii* Td1 grown in skyr at 8 °C for 21 days. Mean values and SD from triplicate experiments are presented.

Yeasts isolates from skyr were identified as *K. marxianus*, *P. kudriavzevii* and *T. delbrueckii* based on the 26S rRNA gene sequencing (Table 1). In addition, sequencing of the 5.8S-ITS region confirmed the strain annotation to *K. marxianus* (100% identity). Only minor differences in the chromosomal length polymorphism were observed for the strains Km1 and Km2, which could be easily separated from the strain Km3 (Supplementary Figure S1). Based on the PFGE profiles, all three dairy strains were, as expected, more closely related to the type strain of *K. marxianus* CBS1553 than to the type strain of *K. lactis* CBS845. All yeast strains were able to grow in skyr at 8 °C, reaching the stationary phase after 7 days of incubation (Figure 1). The highest counts, after 21 days of incubation, were observed for the *K. marxianus* strains (6.95–7.17 Log CFU·g^−1^), while *P. kudriavzevii* Pk1 and *T. delbrueckii* Td1 were grown to lower numbers (5.41 ± 0.15 and 5.65 ± 0.11 Log CFU·g^−1^, respectively). Viable counts of LAB in skyr were stable within the incubation period (9.55–9.74 Log CFU·g^−1^) and were not affected by yeasts. Similarly, pH was basically maintained during the storage period, showing a slight tendency to decrease from 4.16 ± 0.04 (Day 0) to 4.02 ± 0.03 (Day 21).

### 3.2. Production of Volatile Compounds

**Figure 2:**
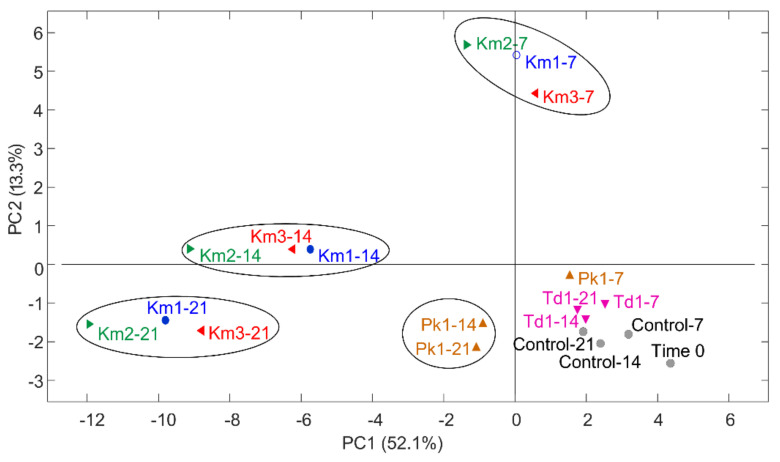
Principal component analysis (PCA) of volatile organic compound (VOC) dataset presented by a score plot of the first two PCs. VOCs were quantified during growth of yeasts *K*. *marxianus* (strains Km1, Km2 and Km3), *P. kudriavzevii* Pk1 and *T. delbrueckii* Td1 in skyr at 8 °C for 21 days, and in controls (skyr without yeasts). Sample codes denote the strain ID and the incubation period (0, 7, 14 and 21 days). Mean values from triplicate experiments were used as input in PCA.

In total, 40 volatile aroma compounds of the main VOC types, including alcohols (12 compounds), aldehydes (eight compounds), esters (14 compounds) and ketones (six compounds), were identified in skyr during the growth of yeasts. The PCA plots (Figure 2) describe variation between the yeasts and incubation time (0, 7, 14 and 21 days) in VOC production. The first two components (PC1 and PC2) explained 65.4% of the variation in the data. Yeasts strains of *K. marxianus* (Km1, Km2 and Km3) were grouped together according to the incubation time and clearly discriminated from the controls. A separate group comprised *P. kudriavzevii* Pk1 samples after 14 and 21 days of incubation. Samples of *T. delbrueckii* Td1 were poorly distinguished from skyr controls, indicating similar VOC patterns.

**Figure 3:**
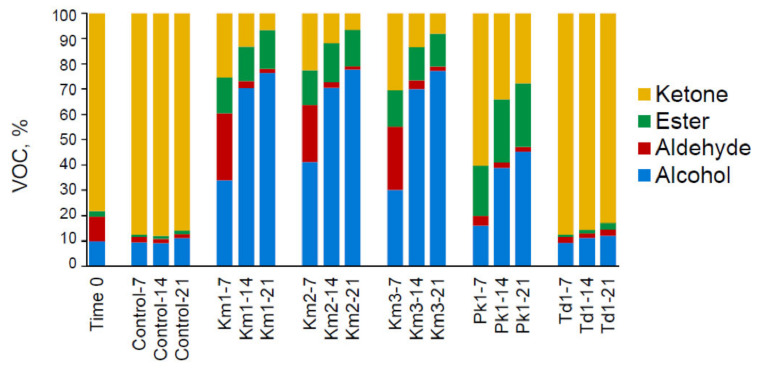
Distribution of the major types of volatile compounds (alcohols, aldehydes, esters and ketones) produced by yeasts *K*. *marxianus* (strains Km1, Km2 and Km3), *P. kudriavzevii* Pk1 and *T. delbrueckii* Td1 grown in skyr at 8 °C for 21 days and in controls (skyr without yeasts). Sample codes denote the strain ID and the incubation period (0, 7, 14 and 21 days). Mean values from triplicate experiments are presented.

Figure 3 presents the relative distribution of the VOC types, calculated by summing the averages of the relevant compounds at 0, 7, 14 and 21 days of incubation. The proportions of alcohols (10% at baseline) were gradually increased during the growth of *K. marxianus* strains (up to 76–78%) and *P. kudriavzevii* Pk1 (up to 45%), along with the amounts of esters (from 2% at baseline to 13–14% and 25%, respectively). Ketones, comprising highly abundant group at the start of incubation (80%), declined to 7–8% for *K. marxianus* strains and to 28% for *P. kudriavzevii* Pk1 at the end of incubation. The initial levels of aldehydes (7–9%) were increased after 7 days incubation of *K. marxianus* strains to 22–26%, followed by a decrease to 2% (Day 21), while for *P. kudriavzevii* Pk1, a gradual decrease in aldehydes was observed (to 2%). Incubation of *T. delbrueckii* Td1 led, primarily, to a slight increase in ketones (by 3–8%) on the account of aldehydes.

**Figure 4:**
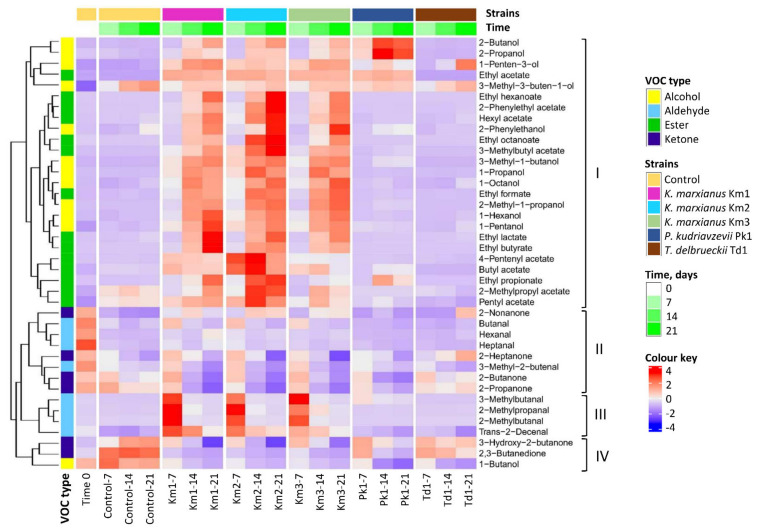
Hierarchical cluster analysis (Pearson’s correlation) and a heat map of VOC profiles shown in relation to yeast strains and incubation time. VOCs were determined during growth of yeasts *K*. *marxianus* (strains Km1, Km2 and Km3), *P. kudriavzevii* Pk1 and *T. delbrueckii* Td1 in skyr at 8 °C for 21 days and in controls (skyr without yeasts). Sample codes denote the strain ID and the incubation period (0, 7, 14 and 21 days). Heat map was constructed using mean values from triplicates.

Concentration of the individual aroma compounds at the start and end of incubation (Day 0 and Day 21), retention indices and odour thresholds in water are shown in Table 2. The results of VOC quantification at Day 7 and 14 are listed in Supplementary Materials (Tables S1 and S2). Figure 4 shows the heatmap and hierarchical cluster analysis of the total VOC data. Alcohols and esters comprised the major VOC cluster in the heatmap (Cluster I, Figure 4). Most of the alcohols were highly increased (more than 10-fold at Day 21) for *K. marxianus* strains compared to controls, including 2-butanol (from 2.1 to 17–21 µg·kg^−1^), 1-hexanol (from 4.5 to 46–56 µg·kg^−1^), 3-methyl-1-butanol or isoamylalcohol (from 21 to 1780–1918 µg·kg^−1^), 2-methyl-1-propanol (from 0.6 to 446–591 µg·kg^−1^), 1-propanol (from 0.4 to 14–15 µg·kg^−1^) and 2-phenylethanol (from 0.4 to 3.2–4.7 µg·kg^−1^) (Table 2). Other alcohols (1-pentanol, 1-penten-3-ol, 1-octanol) were moderately increased (four- to nine-fold) for *K. marxianus* strains. The levels of 3-methyl-1-butanol and 1-pentanol were close to the threshold values. Along with the alcohols, ethyl esters of formic, lactic, propionic and fatty acids, as well as 3-methylbutyl acetate (isoamylacetate) and other acetic acid esters (ethyl-, hexyl, pentenyl etc.), were produced in significantly higher amounts by *K. marxianus* (Table 2, Tables S1 and S2). The major increases above the thresholds were recorded for ethyl acetate (from 0 to 278–291 µg·kg^−1^), ethyl butyrate (from 0.51 to 34–81 µg·kg^−1^), ethyl hexanoate (from 0.1 to 19–32 µg·kg^−1^), ethyl octanoate (from 0.6 to 10–23 µg·kg^−1^), and 3-methylbutyl acetate (from 0.2 to 29–57 µg·kg^−1^) (Table 2). Cluster II (Figure 4) was primarily composed of ketones (2-heptanone, 2-butanone and 2-propanone) and straight-chain aldehydes (butanal, heptanal and hexanal), which were present at high concentration at Day 0 and generally decreased during the incubation of *K. marxianus* and controls. At the end of the incubation period, 2-butanone and 2-propanone (acetone) were found in significantly lower amounts for the *K. marxianus* strains (between 40–62 µg·kg^−1^ and 8–19 µg·kg^−1^, respectively) compared to controls (136 µg·kg^−1^ and 83 µg·kg^−1^, respectively), while 2-nonanone was preserved in *K. marxianus* samples. Cluster III combined branched-chain aldehydes (2-methylbutanal, 3-methylbutanal and 2-methylpropanal), which were dramatically increased during the exponential growth of *K. marxianus* strains (Figure 4 and Table S1). The highest increase was recorded for 2-methylbutanal and 2-methylpropanal (from 5–7 µg·kg^−1^ at Day 0 to 237–283 µg·kg^−1^ and 156–231 µg·kg^−1^, respectively, at Day 7). The levels of these compounds at Day 21 were still two- to four-fold higher than in the controls and close to the threshold values (Table 2). Cluster IV ketones, 2,3-butanedione (diacetyl) and 3-hydroxy-2-butanone (acetoin) were decreased during the incubation of *K. marxianus* strains (from 178 to 48–49 µg·kg^−1^ and from 152 to 55–79 µg·kg^−1^, respectively), whereas they were accumulated in skyr controls.

Several differences in VOC production were observed between the *K. marxianus* strains. Thus, Km1 differed from other strains by the high production of ethyl butyrate, pentanol, hexanol and hexanal. Strain Km2 was associated with increased levels of several esters (ethyl hexanoate, ethyl octanoate and 2-phenylethyl acetate), while Km3 was distinguished by the amounts of 2-methyl propanal (highest) and ethyl propionate (lowest). Growth of *P. kudriavzevii* Pk1 was associated with the increased production of 2-butanol, 3-methyl-1-butanol, 2-propanol, ethyl propionate and ethyl acetate, together with decreases in ketones (2,3-butanedione, 2-butanone and 2-propanone). Only a few significant shifts in VOC production were observed for *T. delbrueckii* Td1, including increases in 1-penten-3-ol and 2-nonanone, as well as decreases in 1-butanol, 3-methyl-2-butenal and 2,3-butanedione, compared to controls (Table 2).

### 3.3. Utilization of Lactose and Galactose

**Figure 5:**
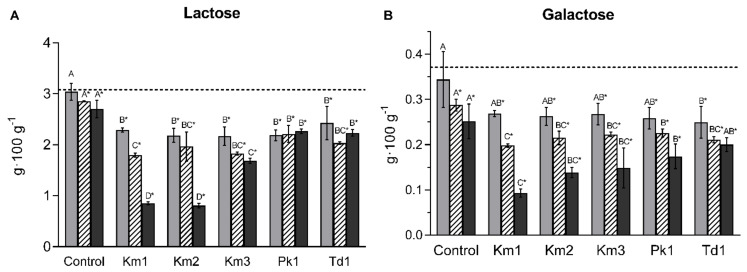
Concentration of lactose (**A**) and galactose (**B**) in skyr after 7 days (grey columns), 14 days (white columns) and 21 days (black columns) incubation of yeasts *K. marxianus* (strains Km1, Km2 and Km3), *P. kudriavzevii* Pk1 and *T. delbrueckii* Td1 at 8 °C, and in controls (skyr without yeasts). The dashed lines show initial concentration of lactose and galactose in skyr (mean values at Day 0). Mean values and SD (bars) from triplicate experiments are presented. Different subscripts denote statistical differences (*p* < 0.05) between the yeast strains within the same period of incubation determined by the one-way ANOVA (Tukey’s post-hoc analysis). Asterisks indicate values significantly different from Day 0 (Student’s *t*-test, *p* < 0.05).

The amount of lactose in skyr (initially 3.10 ± 0.16 g·100 g^−1^) was gradually decreased during incubation of *K. marxianus* strains (Figure 5A). The lowest concentration of lactose was found for Km1 and Km2 (0.84 ± 0.03 and 0.95 ± 0.03 g·100 g^−1^, respectively, at Day 21). Lactose concentration for *P. kudriavzevii* Pk1 and *T. delbrueckii* Td1 was reduced at Day 7 and remained stable afterwards (2.26 ± 0.16 and 2.16 ± 0.28 g·100 g^−1^ at Day 21, respectively; Figure 5A). Concurrently, the levels of galactose (initially 0.37 ± 0.02 g·100 g^−1^) were reduced in yeast incubations and controls, largest for *K. marxianus* Km1 (0.09 ± 0.01 g/100 g^−1^ at Day 21; Figure 5B). The concentration of glucose was negligible and not detected by the applied HPLC. Phenotypic tests showed that *K. marxianus* strains had an ability to ferment and assimilate lactose and galactose, in contrast to *P. kudriavzevii* Pk1 and *T. delbrueckii* Td1 (Table S3).

### 3.4. Production of Organic Acids, Ethanol and Carbon Dioxide

Table 3 shows the amounts of organic acids in skyr during yeast incubation. The major acids at Day 0 were lactic (224 ± 8.5 mg·100 g^−1^), citric (120 ± 9.1 mg·100 g^−1^) and acetic (54 ± 1.4 mg·100 g^−1^) acids. Additionally, orotic and uric acids were detected in lower amounts (5.1 ± 0.16 mg·100 g^−1^ and 3.7 ± 0.36 mg/100 g^−1^, respectively). The levels of lactic acid were generally increased by yeasts and in controls (257–285 mg·100 g^−1^ at Day 21). Citric acid was significantly reduced by yeasts already after 7 days incubation (73–86 mg·100 g^−1^) and remained stable afterwards, except for *K. marxianus* Km1 (further decrease to 48 ± 8.1 mg·100 g^−1^ at Day 21). Similarly, orotic acid was mostly decreased by yeasts, largest by Km1 (3.3 ± 0.14 mg·100 g^−1^ at day 21), while the levels of uric acid were generally stable over time. The reduction in citric and orotic acids, though to a lesser extent, was also observed in skyr controls. Differences between the yeast species were found for acetic acid at Day 21, which was decreased for *K. marxianus* Km1 and Km2 (48 ± 2.4 and 50.4 ± 2.5 mg·100 g^−1^, respectively), opposite to *P. kudriavzevii* Pk1 and *T. delbrueckii* Td1 (increased to 60.2 ± 2.9 and 58 ± 4.1 mg·100 g^−1^, respectively).

**Figure 6:**
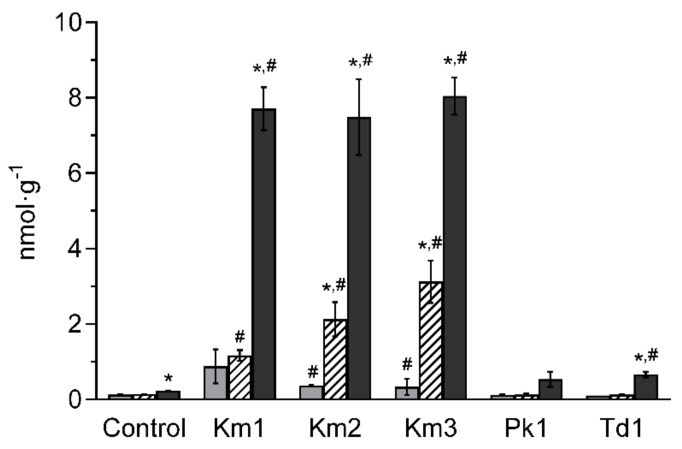
Production of carbon dioxide (CO_2_, nmol·g^−1^) by yeasts *K. marxianus* (strains Km1, Km2 and Km3), *P. kudriavzevii* Pk1 and *T. delbrueckii* Td1 in skyr, and in controls (without addition of yeasts) after 7 days (grey columns), 14 days (white columns) and 21 days (black columns) incubation at 8 °C. Mean values and SD (bars) from triplicate experiments are presented. Hashes denote significant differences between yeasts and controls within the same day of incubation, and asterisks indicate significant differences across the time points determined by the Student’s *t*-test (*p* < 0.05).

Ethanol was detected for *K. marxianus* at Day 21 in a range of 0.11–0.13%; other samples tested negative for ethanol. Carbon dioxide was continuously produced by *K. marxianus* Km1, Km2 and Km3, reaching concentrations of 7.20 ± 1.26, 7.49 ± 1.01 and 8.30 ± 0.31 nmol·g^−1^, respectively, at Day 21 (Figure 6). Production of CO_2_ by *P. kudriavzevii* Pk1 and *T. delbrueckii* Td1 was comparatively low (0.60 ± 0.25 and 0.69 ± 0.09 nmol·g^−1^ at Day 21, respectively).

## 4. Discussion

This study investigated the spoilage activity of the yeasts *K. marxianus* (Km1, Km2 and Km3), *P. kudriavzevii* Pk1 and *T. delbrueckii* Td1, proliferating in skyr in cold storage. We were particularly focused on the most common spoilage species *K. marxianus*, of which strains were isolated from skyr and identified in this study. As expected, all strains of *K. marxianus* were able to grow in skyr to the levels close to those reported for yoghurts (6–8 Log CFU·g^−1^) at refrigerated temperatures [22,35,36]. High counts of *K. marxianus* were due to the ability of this species to ferment lactose and galactose in skyr, with the production of CO_2_ and ethanol, promoted by oxygen limitation conditions. These results are consistent with earlier studies on yoghurts [36,37,38]. In contrast to *K. marxianus*, *P. kudriavzevii* Pk1 and *T. delbrueckii* Td1 were unable to utilize lactose and galactose, leading to poorer growth and metabolite release.

The flavour of skyr, likewise with other fermented milk products, is created by a balanced mixture of various essential VOCs. It is well recognized that the shifts in VOC amounts, rather than the absence or presence of a particular compound, give rise to off-flavours in dairy products during storage [39,40]. In this study, VOC profiles in skyr highly depended on the yeast species and incubation time. The absolutely largest diversity and changes in VOCs were observed for the strains of *K. marxianus*, in accordance with their capacity to grow in skyr to high numbers. The impact of *P. kudriavzevii* Pk1 on individual VOCs was less pronounced, while only minor significant differences from controls were recorded for *T. delbrueckii* Td1, indicating that the spoilage potential is a combination of both cell numbers and the off-flavour profile. The following discussion will focus mostly on VOC production by *K. marxianus* in relation to the undesired aroma and spoilage potential.

Higher (fusel) alcohols were accumulated during *K. marxianus* propagation in skyr, comprising a major VOC fraction. Higher alcohols are produced by yeasts via the Ehrlich pathways through conversion of α-keto acids into aldehydes, which can be either further reduced to higher alcohols or dehydrogenated to organic acids [41]. The activity of the Ehrlich pathways is known to be growth-phase dependent [41,42]. Accordingly, shifts in the levels of aldehydes were recorded at the exponential growth phase of *K. marxianus* (0–7 days). A reduction in the straight chain aldehydes and temporary increase in the branched chain aldehydes was accompanied by an increase in respective alcohols (e.g., 1-hexanol, 3-methyl-1-butanol and 2-methyl-1-propanol). The increase in the alcohols continued through the stationary growth (14–21 days), indicating that the conversion of aldehydes into alcohols was still active.

The high production of fusel alcohols indicated fermentative dissimilation of lactose by *K. marxianus*, taking place at oxygen limitation conditions. Generally, alcohols have lower odour activity compared to aldehydes and are considered to be of less importance for yoghurt aroma [5,43]. The individual alcohols in this study were detected in amounts lower than the thresholds (100–40,000 µg·kg^−1^). Despite this, the impact of alcohols on the overall skyr aroma should not be undervalued, considering their considerable accumulative levels in *K. marxianus* samples. Specifically, alcohols with relatively low thresholds, such as highly abundant isoamylalcohol (whiskey, malt aroma) and 2-methyl-1-propanol (alcoholic aroma), as well as the less abundant 1-pentanol (fusel) and 1-octanol (orange), might contribute to the off-flavours in skyr. Supporting this assumption, Güler reported a correlation between increases in isoamylalcohol and 2-methyl-1-propanol with a flavour decline in salted yogurt during storage [44]. One of the functions of the Ehrlich pathways is to form quorum-sensing compounds that can induce differentiation and participate in the adaptation of yeast cells to environmental changes [41]. Alcohol 2-phenylethanol, produced by *K. marxianus* in moderate amounts, is the most well-studied quorum-sensing molecule in dimorphic yeasts (e.g., *K. marxianus* and *S. cerevisiae*) [45,46]. Balbino et al. showed that exposure of *K. marxianus* to 2-phenylethanol provoked changes in cell morphology and fermentative metabolism, and impaired yeast viability [47]. Isoamylalcohol is another signalling compound, which has been reported to induce pseudohyphal growth and filamentation in *S. cerevisiae* [48]. Regarding the major release of isoamylalcohol by *K. marxianus* in this study, the significance of this compound and 2-phenylethanol for yeast growth and differentiation during skyr spoilage needs to be further examined.

Aldehydes, even at low levels, may affect a product’s flavour due to their low threshold values. The branched chain aldehydes, methylbutanals (apple-like, malt aroma) and 2-methylpropanal (sharp, pungent aroma), along with hexanal (sharp, fruity aroma) and heptanal (fatty, pungent aroma) would probably account for the initial off-flavours in skyr contaminated with *K. marxianus*. The same compounds have been referred as primary sources of undesired malty, nutty flavours in cheese [49,50,51]. Carrillo-Carrión and co-workers proposed that the total concentration of volatile aldehydes (C5-C9) can be used as a marker of yogurt degradation at inadequate storage conditions [52]. Among them, hexanal was positively associated with rancidity in foods and negatively associated with the “degree of likeness” in yoghurts [53,54].

Esters composed the second largest group of aroma compounds produced by *K. marxianus*. Esters are formed by a condensation reaction between alcohols (ethanol or higher alcohols) and acetyl-CoA (for acetate esters) or acyl-CoA (for ethyl esters) [55,56]. It is well known that ester production is positively affected by precursor availability, i.e., the alcohols released by *K. marxianus* in this study, and the presence of lactose in skyr required for acetyl-CoA formation [43,57]. Esters are characterized by relatively low threshold values and typically impart a fruity aroma to the dairy products [43,44,58]. In this study, ethyl acetate (solvent aroma), ethyl butyrate (pineapple), ethyl hexanoate (aniseed, apple), ethyl octanoate (sour apple) and isoamylacetate (banana, pineapple) were produced in large quantities, close to or higher than the thresholds. The ability of *K. marxianus* strains to produce esters, in particular ethyl acetate, has been frequently reported [38,56,59,60]. Previously, Leclercq-Perlat et al. identified similar aroma compounds produced by *K. marxianus* in a cheese medium (e.g., hexanal, 2 (3)-methylbutanal, butanol, 2 (3)-methylbutanol, 2-methylpropanol) and reported a link between the levels of isoamylacetate and ethyl acetate, and ester-like fruity odours [61].

The vicinal ketones diacetyl and acetoin, together with methyl ketones 2-butanone and 2-propanone, were mostly decreased by *K. marxianus* strains and *P. kudriavzevii* Pk1, in contrast to the skyr controls (accumulated or maintained). These ketones have been referred to as the key aroma compounds, associated with butter-like, creamy and fruity flavours in yoghurts [7,53,62,63]. The diacetyl flavour is particularly important due to a low threshold value [5,64]. Diacetyl can be produced by lactic acid bacteria and yeasts from lactose by oxidative decarboxylation of α-acetolactate, and further converted to acetoin by intracellular reductases [5,65]. Regarding the low reductase activity reported for the starter cultures *S. thermophilus* and *L. bulgaricus*, accumulation of diacetyl and acetoin in skyr was probably enhanced by the metabolic activity of *L. paracasei* (bioprotective culture in skyr) [66,67]. Lower levels of diacetyl and acetoin for the *K. marxianus* strains and *P. kudriavzevii* Pk1 can be explained by the redirection of pyruvate metabolism towards the production of carbon dioxide and ethanol, and/or towards the Ehrlich pathways [65]. Reduction in methyl ketones in yeast fermentations might be due to their conversion to the corresponding alcohols (2-propanol and 2-butanol). Furthermore, the presence of lactose, an easily assimilated carbon source, might result in repression of lipid oxidation and, consequently, the production of methyl ketones [61].

Different strains of *K. marxianus* exhibited, more or less, similar VOC patterns with a few variations in specific compounds. Regarding these variations, *K. marxianus* Km1 is expected to be associated with more fusel alcohol and a pungent flavour (higher release of 1-hexanol, 1-pentanol, and ethyl butyrate), while *K. marxianus* Km2 will probably impart a hint of fruity apple-like odours (larger amounts of ethyl hexanoate and ethyl octanoate). Off-flavours related to *P. kudriavzevii* Pk1 may occur from increases in isoamyl acetate and ethyl acetate (both higher than their threshold values), along with a decline in ketones and ethyl esters. Although changes in VOCs for *T. delbrueckii* Td1 were mostly insignificant, the combined impact, e.g., as shifts in essential ketones, diacetyl, 2-heptanone and 2-nonanone, should not be excluded. It should be noted, however, that thresholds in this study has been reported in water and would therefore differ from the actual values, since the main components in skyr (carbohydrates, proteins and fat) would counteract VOC release [49,68,69]. Thus, further sensory evaluation of aroma perception in skyr is needed to draw conclusions of yeast-related off-flavours.

Organic acids contribute to the sensory characteristics and act as preservatives in fermented milk products [70,71,72]. Citric acid or citrate is normally present in raw milk in high amounts and can be degraded by LAB to acetate, lactate, ethanol and acetoin as the major end products [4,73]. The largest reduction in citric acid was observed in yeast incubations, especially for *K. marxianus* Km1, indicating strain-specific differences in citrate utilization. These results are in accordance with previous findings, showing the ability of some *K. marxianus* strains to metabolize citric acid [74,75]. Acetic acid is produced from pyruvate by LAB and yeasts via heterolactic fermentation pathways [73]. In accordance with VOC analysis, lower amounts of acetic acid in *K. marxianus* incubations can be related to acetate conversion into esters, and/or its utilization as a carbon source for the production of ethanol and carbon dioxide. Orotic acid is an intermediate compound in pyrimidine biosynthetic pathways that can be used as a growth factor by the starter cultures [70,76]. Thus, a reduction in orotic acid by yeasts in the exponential growth phase indicated the activation of nucleotide biosynthesis essential for cell growth. Similar to this study, a minor decrease in orotic acid has been reported in yoghurts in cold storage [76,77,78].

## 5. Conclusions

Spoilage yeasts *K. marxianus* (strains Km1, Km2 and Km3), *P. kudriavzevii* Pk1 and *T. delbrueckii* Td1 were able to proliferate in skyr during refrigerated storage, exhibiting species-specific growth characteristics and metabolite production. Strains of *K. marxianus* were characterized by the largest viable counts and major release of various aroma compounds. Many of the VOCs, produced in amounts higher than the threshold values, were previously related to off-flavours and product deterioration. In contrast to the other species, the growth of *K. marxianus* in skyr resulted in the production of carbon dioxide and ethanol. Extensive growth capacity, and the formation of undesired aroma and carbon dioxide indicated a high spoilage potential of *K. marxianus* contaminants. The impact of other yeast species on skyr aroma was less pronounced. In particular, *T. delbrueckii* Td1 had low spoilage activity, showing only minor changes in metabolite profiles, as compared to non-contaminated skyr. The current study clarifies metabolic activities in yeast species, associated with the spoilage of skyr, and highlights the importance of distinguishing between contaminating yeast species, and even strains, when evaluating their impact on product quality and shelf-life. A paradigm shift in yeast quality control of fermented milk products is recommended, moving from solely determining colony forming units towards more detailed studies, focusing on yeast species identification, growth patterns and metabolite profiles.

## Figures and Tables

**Table 1:** Yeasts in this study identified by 26S rRNA gene sequencing.

Species	Strain ID	Identity, %	GeneBank Accession nr.	Origin
*Kluyveromyces marxianus*	Km1	100	OM854769	This study
*Kluyveromyces marxianus*	Km2	100	OM854770	This study
*Kluyveromyces marxianus*	Km3	99.8	OM854771	This study
*Pichia kudriavzevii*	Pk1	100	OM854772	Arla Foods, Denmark
*Torulaspora delbrueckii*	Td1	100	OM854773	Arla Foods, Denmark

**Table 2:** Concentration of volatile compounds in skyr at start (VOC_t0_) and after 21 days (VOC_t21_) incubation of yeasts *Kluyveromyces marxianus* Km1 Km2 and Km3, *Pichia kudriavzevii* Pk1 and *Torulaspora delbrueckii* Td1, and in skyr controls (without added yeasts), VOC retention indices (RI) and threshold values.

Compound	RI Calculated	RI ^1^ Reference	VOC_t0_, µg·kg^−1 3^	VOC_t21_, µg·kg^−1 2^						Odour Threshold µg·kg^−1 3^	Odour Description ^4^
				Control	*K. marxianus* Km1	*K. marxianus* Km2	*K. marxianus* Km3	*P. kudriavzevii* Pk1	*T. delbrueckii* Td1		
**Alcohols**											
1-Butanol	1166	1166	37 ± 6.9	39 ± 1.4 ^A^	22 ± 1.9 ^B^	23 ± 1.9 ^B^	23 ± 5.1 ^B^	14 ± 4.3 ^B^	19 ± 2.7 ^B^	500-2500	rancid, sweet
2-Butanol	1031	988–1053	2.1 ± 0.5	1.6 ± 0.05 ^B^	19 ± 3.2 ^A^	21 ± 0.62 ^A^	17 ± 3.4 ^A^	28 ± 7.9 ^A^	1.6 ± 0.17 ^B^	3300	vinous, fruity
1-Hexanol	1355	1372	3.8 ± 1.0	4.5 ± 1.4 ^C^	56 ± 3.1 ^A^	46 ± 2.4 ^B^	48 ± 2.1 ^B^	4.8 ± 0.53 ^C^	5.9 ± 1.3 ^C^	2500	sweet alcohol
3-Methyl-1-butanol	1227	1174–1255	21 ± 4.9	34 ± 13 ^C^	1780 ± 48 ^A^	1918 ± 100 ^A^	1781 ± 163 ^A^	412 ± 63 ^B^	30 ± 5 ^C^	3060	whiskey, malt, burnt
3-Methyl-3-buten-1-ol	1262	1226–1277	10.1 ± 2.4	32 ± 1.8	26 ± 1.2	27 ± 1.6	26 ± 3.4	25 ± 2.4	28 ± 2.6	250	sweet fruity
2-Methyl-1-propanol	1110	1110	0.61 ± 0.25	1.4 ± 0.62 ^B^	446 ± 58 ^A^	562 ± 17 ^A^	591 ± 102 ^A^	38 ± 8.9 ^AB^	0.90 ± 0.15 ^B^	40,000	sweet musty, fusel
1-Pentanol	1267	1273	6.5 ± 1.6	8.1 ± 0.68 ^C^	30 ± 0.76 ^A^	24 ± 2.1 ^B^	23 ± 2.4 ^B^	9.2 ± 0.85 ^C^	9.9 ± 1.7 ^C^	120	fusel
1-Penten-3-ol	1179	1177	0.28 ± 0.12	0.40 ± 0.15 ^B^	2.2 ± 0.15 ^A^	1.4 ± 0.19 ^AB^	1.9 ± 0.36 ^A^	0.99 ± 0.25 ^AB^	2.3 ± 0.83 ^A^	500	pungent, fruity
2-Phenylethanol	1923	1930	0.36 ± 0.16	1.1 ± 1.04 ^BC^	3.2 ± 0.20 ^AB^	4.7 ± 0.41 ^A^	4.5 ± 0.80 ^A^	1.04 ± 0.71 ^BC^	0.36 ± 0.41 ^C^	750–1100	mild rose
1-Propanol	1043	1041	0.35 ± 0.08	0.35 ± 0.05 ^B^	14 ± 3.4 ^A^	15 ± 2.3 ^A^	14 ± 2.02 ^A^	0.69 ± 0.01 ^B^	0.34 ± 0.06 ^B^	9000	alcoholic
2-Propanol	936	884–963	1.1 ± 0.20	0.65 ± 0.41 ^B^	2.7 ± 0.81 ^AB^	3.3 ± 1.3 ^AB^	3.5 ± 0.53 ^AB^	6.9 ± 4.01 ^A^	0.68 ± 0.06 ^B^	40,000-78,000	rubbing alcohol
1-Octanol	1559	1570	5.4 ± 1.7	5.8 ± 2.4 ^C^	15 ± 0.80 ^B^	20 ± 1.7 ^AB^	21 ± 1.4 ^A^	4.8 ± 0.44 ^C^	7.0 ± 1.9 ^C^	110–130	aromatic, orange
**Aldehydes**											
Butanal	875	830–911	2.9 ± 0.92	0.82 ± 0.17	0.59 ± 0.13	0.45 ± 0.02	0.60 ± 0.15	0.33 ± 0.15	0.70 ± 0.17	7–37	pungent
Heptanal	1191	1189	12 ± 3.3	1.1 ± 0.14 ^B^	3.9 ± 1.1 ^A^	2.5 ± 0.22 ^AB^	2.3 ± 0.63 ^AB^	0.76 ± 0.10 ^B^	1.1 ± 0.34 ^B^	3–60	fatty, pungent, fruity
Hexanal	1079	1082	21 ± 5.7	1.3 ± 1.1 ^B^	8.6 ± 0.55 ^A^	4.2 ± 0.22 ^AB^	3.8 ± 0.79 ^B^	0.64 ± 0.07 ^B^	2.7 ± 1.9 ^B^	5–9	sharp, fruity, grass
2-Methylbutanal	912	912	4.9 ± 1.1	3.3 ± 0.07 ^B^	10.5 ± 0.72 ^A^	11 ± 1.7 ^A^	14 ± 3.5 ^A^	0.35 ± 0.33 ^B^	3.6 ± 0.34 ^B^	1–6	apple, malt
3-Methylbutanal	916	916	7.6 ± 1.9	2.9 ± 1.0 ^B^	9.3 ± 0.45 ^A^	10.2 ± 0.75 ^A^	9.8 ± 1.8 ^A^	12 ± 2.3 ^A^	4.03 ± 0.24 ^B^	1–3	apple, malt
3-Methyl-2-butenal	1199	1189–1236	6.8 ± 1.5	2.5 ± 0.45 ^A^	0.46 ± 0.01 ^C^	0.66 ± 0.07 ^C^	0.67 ± 0.15 ^C^	2.1 ± 0.30 ^AB^	1.3 ± 0.42 ^BC^	nr	metallic, herbaceous
2-Methylpropanal	811	812	7.1 ± 1.1	5.3 ± 0.53 ^D^	10.2 ± 1.6 ^BC^	12 ± 0.32 ^B^	19 ± 2.2 ^A^	5.4 ± 0.90 ^D^	6.2 ± 0.70 ^CD^	1.5	sharp, pungent
Trans-2-Decenal	1660	1662	4.9 ± 1.7	2.9 ± 2.3 ^AB^	5.3 ± 0.33 ^AB^	6.9 ± 0.55 ^A^	6.8 ± 1.3 ^A^	1.9 ± 0.28 ^B^	3.2 ± 1.3 ^AB^	0.4	orange, tallow
**Esters**											
Butyl acetate	1070	1082	0.019 ± 0.036	0.51 ± 0.10 ^AB^	1.8 ± 0.71 ^AB^	2.8 ± 1.6 ^A^	0.92 ± 0.15 ^AB^	1.01 ± 0.60 ^AB^	0.094 ± 0.023 ^B^	10–100	sweet, fruity
Ethyl acetate	891	891	nd	nd ^B^	289 ± 4.7 ^A^	283 ± 15 ^A^	291 ± 0.8 ^A^	278 ± 15 ^A^	nd^B^	5–5000	pineapple, solvent
Ethyl butyrate	1033	1036	0.51 ± 0.15	0.87 ± 0.23 ^D^	81 ± 4.2 ^A^	44 ± 2.2 ^B^	34 ± 2.3 ^C^	1.63 ± 0.48 ^D^	0.57 ± 0.06 ^D^	1	pineapple
Ethyl hexanoate	1244	1251	0.093 ± 0.039	0.072 ± 0.077 ^C^	23 ± 2.1 ^B^	32 ± 1.5 ^A^	19 ± 1.2 ^B^	nd ^C^	0.18 ± 0.06 ^C^	1	fruity, aniseed, apple
Ethyl octanoate	1419	1445	0.55 ± 0.24	0.26 ± 0.37 ^C^	10.1 ± 1.2 ^B^	23 ± 5.7 ^A^	14 ± 2.1 ^B^	nd ^C^	0.11 ± 0.15 ^C^	15	fruity, sour apple
Ethyl formate	823	823	0.27 ± 0.17	0.038 ± 0.054 ^B^	5.7 ± 2.9 ^A^	7.5 ± 0.36 ^A^	8.5 ± 1.5 ^A^	0.39 ± 0.08 ^B^	0.27 ± 0.04 ^B^	150,000	fruity, pungent
Ethyl lactate	1344	1356	0.20 ± 0.07	1.3 ± 0.89 ^C^	21 ± 1.3 ^A^	17 ± 1.7 ^AB^	14 ± 2.4 ^B^	0.58 ± 0.26 ^C^	0.82 ± 0.68 ^C^	14,000	buttery, fruity
Ethyl propionate	956	956	nd	0.076 ± 0.069 ^B^	6.2 ± 2.6 ^A^	7.6 ± 4.9 ^AB^	1.3 ± 0.15 ^B^	2.1 ± 1.1 ^AB^	nd ^B^	10	fruity, rum, pineapple
Hexyl acetate	1281	1291	0.13 ± 0.04	0.26 ± 0.04 ^B^	1.3 ± 0.23 ^A^	1.7 ± 0.30 ^A^	1.2 ± 0.12 ^A^	0.21 ± 0.01 ^B^	0.24 ± 0.04 ^B^	2	sweet, fruity, pear
2-Methylpropyl acetate	1013	1018	0.011 ± 0.007	1.78 ± 0.33	3.6 ± 1.3	5.1 ± 3.5	2.1 ± 0.12	0.87 ± 1.01	0.33 ± 0.14	66	fruity, floral
3-Methylbutyl acetate	1136	1142	0.17 ± 0.091	0.30 ± 0.31 ^C^	33 ± 8.1 ^AB^	57 ± 21 ^A^	29 ± 0.49 ^AB^	3.8 ± 2.02 ^BC^	0.31 ± 0.31 ^C^	5	sweet, fruity, banana
4-Pentenyl acetate	1199	1204	0.078 ± 0.036	0.08 ± 0.02 ^B^	2.08 ± 1.04 ^AB^	3.6 ± 2.4 ^A^	0.92 ± 0.36 ^AB^	0.13 ± 0.11 ^B^	0.01 ± 0.01 ^B^	nr	green, plastic, weedy
Pentyl acetate	1183	1185	0.22 ± 0.11	0.58 ± 0.18 ^AB^	0.85 ± 0.24 ^AB^	0.94 ± 0.31 ^A^	0.59 ± 0.01 ^AB^	0.36 ± 0.12 ^AB^	0.23 ± 0.04 ^B^	80	banana
2-Phenylethyl acetate	1821	1835	0.074 ± 0.041	0.10 ± 0.13 ^C^	2.3 ± 0.44 ^B^	5.03 ± 0.28 ^A^	2.9 ± 0.47 ^B^	0.04 ± 0.05 ^C^	0.10 ± 0.07 ^C^	650	sweet, floral, fruity
**Ketones**											
2,3-Butanedione	984	985	178 ± 36	526 ± 11 ^A^	48 ± 4.3 ^C^	49 ± 10 ^C^	48 ± 5.4 ^C^	72 ± 9.3 ^C^	251 ± 47 ^B^	1.1–6.5	chlorine, buttery
2-Butanone	905	906	191 ± 25	136 ± 5.6 ^A^	40 ± 7.9 ^B^	44 ± 2.9 ^B^	62 ± 11 ^B^	47 ± 11 ^B^	142 ± 10.5 ^A^	8400	ethereal, fruity
2-Heptanone	1189	1189	60 ± 9	46 ± 4.2 ^B^	47 ± 1.4 ^B^	41 ± 1.9 ^B^	40.2 ± 2.7 ^B^	46 ± 0.22 ^B^	60.1 ± 2.6 ^A^	140–3000	fruity, banana, spicy
3-Hydroxy-2-butanone	1290	1302	152 ± 28	201 ± 10 ^A^	55 ± 0.92 ^B^	56 ± 15 ^B^	79 ± 15 ^B^	128 ± 30.3 ^AB^	173 ± 48 ^A^	800	buttery, woody, yogurt
2-Nonanone	1380	1398	31 ± 10	0.41 ± 0.58 ^B^	12 ± 0.80 ^AB^	20.5 ± 1.5 ^A^	18 ± 2.5 ^A^	2.5 ± 0.33 ^B^	24 ± 10.6 ^A^	5-200	hot milk, soap, green
2-Propanone	815	815	109 ± 12	83 ± 8.2 ^A^	8.3 ± 2.9 ^B^	10.4 ± 1.4 ^B^	19 ± 1.5 ^B^	38 ± 28 ^B^	86 ± 8.7 ^A^	20,000	fruity

^1^ Reference RI are from analysis of authentic reference compounds or reported intervals of RI (https://pubchem.ncbi.nlm.nih.gov). ^2^ Relative abundance of each compound was calculated from ratio of the peak area of the compound to the internal standard (4-Methyl-1-pentanol). Different superscript within a raw denote statistically different values (*p* < 0.05) determined by the one-way ANOVA (Tukey’s post-hoc analysis). ^3^ Odour thresholds from https://pubchem.ncbi.nlm.nih.gov, or and http://www.leffingwell.com/odorthre.htm; nr–not recorded. ^4^ Odour description from https://pubchem.ncbi.nlm.nih.gov, or http://www.thegoodscentscompany.com/.

**Table 3:** Concentration of organic acids in skyr during yeast incubation.

Organic Acids	Initial Concentration, mg·100 g^−1^	Yeast Species	Concentration, mg·100 g^−1 1^		
			Day 7	Day 14	Day 21
Acetic acid	54 ± 1.4	Control	57 ± 2.9	57 ± 2.2	67 ± 4.2 ^A^ *
		*K. marxianus* Km1	54 ± 3.2	52 ± 1.1	48 ± 2.4 ^C^ *
		*K. marxianus* Km2	61 ± 4.7	56 ± 0.67 *	50.4 ± 2.5 ^BC^
		*K. marxianus* Km3	56 ± 4.8	54 ± 1.09	58 ± 7.2 ^ABC^
		*P. kudriavzevii* Pk1	58 ± 2.4	54 ± 3.4	60.2 ± 2.9 ^AB^
		*T. delbrueckii* Td1	55 ± 6.3	55 ± 0.94	58 ± 4.1 ^ABC^
Citric acid	120 ± 9.1	Control	94 ± 3.4 ^A^ *	98 ± 13 ^A^	96 ± 5.7 ^A^ *
		*K. marxianus* Km1	86 ± 1.8 ^AB^ *	74 ± 1.1 ^B^ *	48 ± 8.1 ^B^ *
		*K. marxianus* Km2	81 ± 6.9 ^AB^ *	74 ± 1.6 ^B^ *	80 ± 8.4 ^A^ *
		*K. marxianus* Km3	82 ± 7.7 ^AB^ *	74 ± 2.7 ^B^ *	83 ± 7.9 ^A^ *
		*P. kudriavzevii* Pk1	81 ± 7.3 ^AB^ *	81 ± 7.3 ^AB^ *	75 ± 14 ^A^ *
		*T. delbrueckii* Td1	73 ± 3.9 ^B^ *	71 ± 3.1 ^B^ *	79 ± 0.64 ^A^ *
Lactic acid	224 ± 8.5	Control	244 ± 13 ^A^	267 ± 1.4 ^A^ *	287 ± 31 *
		*K. marxianus* Km1	223 ± 4.8 ^AB^	233 ± 3.1 ^B^	257 ± 5.3 *
		*K. marxianus* Km2	218 ± 5.2 ^B^	245 ± 18 ^AB^	261 ± 6.4 *
		*K. marxianus* Km3	219 ± 7.9 ^B^	236 ± 0.57 ^B^	285 ± 19 *
		*P. kudriavzevii* Pk1	223 ± 6.1 ^AB^	241 ± 9.7 ^B^	283 ± 12 *
		*T. delbrueckii* Td1	221 ± 9.2 ^B^	231 ± 1.8 ^B^	271 ± 16 *
Orotic acid	5.1 ± 0.16	Control	4.7 ± 0.38	4.6 ± 0.09 ^A^ *	4.8 ± 0.57 ^A^
		*K. marxianus* Km1	4.3 ± 0.09 *	4.1 ± 0.03 ^B^ *	3.3 ± 0.14 ^B^ *
		*K. marxianus* Km2	4.2 ± 0.17 *	5.1 ± 0.29 ^A^	4.4 ± 0.17 ^A^ *
		*K. marxianus* Km3	4.1 ± 0.19 *	4.1 ± 0.08 ^B^ *	4.8 ± 0.41 ^A^
		*P. kudriavzevii* Pk1	4.2 ± 0.17 *	4.2 ± 0.23 ^B^ *	5.01 ± 0.29 ^A^
		*T. delbrueckii* Td1	4.02 ± 0.39 *	4.1 ± 0.06 ^B^ *	4.6 ± 0.34 ^A^
Uric acid	3.7 ± 0.36	Control	3.1 ± 0.91	3.5 ± 0.20 ^A^	3.7 ± 0.73
		*K. marxianus* Km1	2.7 ± 0.38	2.5 ± 0.20 ^B^ *	2.6 ± 0.19 *
		*K. marxianus* Km2	3.1 ± 0.24	3.4 ± 0.20 ^A^	2.8 ± 0.58
		*K. marxianus* Km3	3.03 ± 0.29	3.2 ± 0.40 ^A^	3.2 ± 0.74
		*P. kudriavzevii* Pk1	3.02 ± 0.40	3.2 ± 0.18 ^A^ *	3.2 ± 0.60
		*T. delbrueckii* Td1	2.9 ± 0.45 *	3.01 ± 0.21 ^AB^	3.5 ± 0.40

^1^ Different subscripts within a column denote values statistically different (*p* < 0.05) between the treatments, as determined by one-way ANOVA and Tukey’s post-hoc analysis. Asterisks denote statistical differences (*p* < 0.05) compared to the initial values (Student’s *t*-test).

## Data Availability

Data is contained within the article or Supplementary Materials.

## Supplementary Materials

The following supporting information can be downloaded at: https://www.mdpi.com/article/10.3390/foods11121776/s1, Figure S1: Dendrograms, showing clustering of *Kluyveromyces marxianus* Km1, Km2, Km3 and the type strains of *Kluyveromyces marxianus* CBS1553 and *Kluyveromyces lactis* CBS845; Table S1: Concentration of volatile compounds (VOC) in skyr after 7 days incubation of yeasts *Kluyveromyces marxianus* Km1, Km2 and Km3, *Pichia kudriavzevii* Pk1 and *Torulaspora delbrueckii* Td1, and in skyr controls (without added yeasts); Table S2 Concentration of volatile compounds (VOC) in skyr after 14 days incubation of yeasts *Kluyveromyces marxianus* Km1, Km2 and Km3, *Pichia kudriavzevii* Pk1 and *Torulaspora delbrueckii* Td1, and in skyr controls (without added yeasts), and Table S3: Fermentation and assimilation of lactose, glucose and galactose by yeasts *Kluyveromyces marxianus* Km1, Km2 and Km3, *Pichia kudriavzevii* Pk1 and *Torulaspora delbrueckii* Td1.
