# Supplementary material for: Spoilage Potential of Contaminating Yeast Species Kluyveromyces marxianus, Pichia kudriavzevii and Torulaspora delbrueckii during Cold Storage of Skyr
Source: Foods. 2022 Jun 16;11(12):1776. doi: 10.3390/foods11121776 (PMC9223127; doi:10.3390/foods11121776)
Supplement: Supplementary file 1 [file foods-11-01776-s001.zip › foods-1754443-supplementary.pdf]

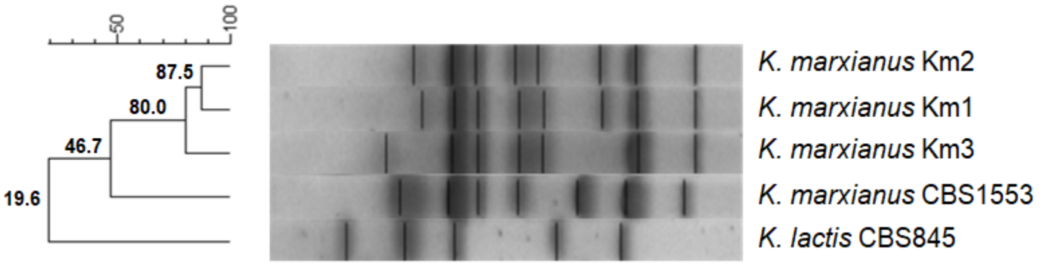

Figure S1. Dendrograms, showing clustering of *Kluyveromyces marxianus* Km1, Km2, Km3 and the type strains of *Kluyveromyces marxianus* CBS1553 and *Kluyveromyces lactis* CBS845. The chromosome length polymorphism of the yeast strains was analysed by pulsed-field gel electrophoresis. Clustering of the chromosomal profiles was performed using the unweighted pair group method with arithmetic averages algorithm and the Dice's coefficient of similarity.

Table S1. Concentration of volatile compounds (VOC) in skyr after 7 days incubation of yeasts *Kluyveromyces marxianus* Km1, Km2 and Km3, *Pichia kudriavzevii* Pk1 and *Torulaspora delbrueckii* Td1, and in skyr controls (without added yeasts).

**Compound**	**VOC at Day 7, $\mu g \cdot kg^{-1}$** [1]					
	**Control**	***K. marxianus* Km1**	***K. marxianus* Km2**	***K. marxianus* Km3**	***P. kudriavzevii* Pk1**	***T. delbrueckii* Td1**
**Alcohols**						
1-Butanol	46 ± 2.8 ^{A}	30 ± 2.1 ^{B}	29 ± 4.1 ^{B}	28 ± 0.76 ^{B}	30 ± 0.07 ^{B}	30 ± 2.5 ^{B}
2-Butanol	1.8 ± 0.18 ^{B}	2.7 ± 0.26 ^{B}	2.8 ± 0.82 ^{B}	2.5 ± 0.31 ^{B}	13 ± 3.1 ^{A}	1.9 ± 0.18 ^{B}
1-Hexanol	2.4 ± 0.42 ^{C}	11 ± 1.5 ^{AB}	16 ± 4.0 ^{A}	12 ± 2.2 ^{A}	5.1 ± 0.33 ^{BC}	4.3 ± 0.65 ^{C}
3-Methyl-1-butanol	22 ± 3.4 ^{C}	701 ± 82 ^{AB}	905 ± 111 ^{A}	553 ± 57 ^{B}	127 ± 24 ^{C}	22 ± 7.6 ^{C}
3-Methyl-3-buten-1-ol	20 ± 2.5	21 ± 0.94	22 ± 2.3	22 ± 0.61	24 ± 0.46	20 ± 1.7
2-Methyl-1-propanol	1.1 ±0.15 ^{C}	60 ± 15 ^{AB}	93 ± 30 ^{A}	31 ± 5.9 ^{BC}	11 ± 3.1 ^{C}	0.58 ± 0.15 ^{C}
1-Pentanol	8.2 ± 1.2 ^{B}	13 ± 0.53 ^{A}	14 ± 0.92 ^{A}	12 ± 0.38 ^{A}	10 ± 0.24 ^{B}	7.7 ± 1.8 ^{B}
1-Penten-3-ol	0.26 ± 0.03 ^{C}	1.4 ± 0.18 ^{A}	1.4 ± 0.17 ^{A}	1.4 ± 0.23 ^{A}	0.87 ± 0.09 ^{B}	0.46 ± 0.14 ^{BC}
2-Phenylethanol	0.04 ± 0.05 ^{B}	0.44 ± 0.22 ^{AB}	0.65 ± 0.14 ^{A}	0.24 ± 0.10 ^{AB}	0.34 ± 0.08 ^{AB}	0.11 ± 0.16 ^{B}
1-Propanol	0.38 ± 0.05 ^{C}	6.5 ± 0.84 ^{A}	7.3 ± 1.2 ^{A}	3.7 ± 0.35 ^{B}	0.49 ± 0.10 ^{C}	0.35 ± 0.07 ^{C}
2-Propanol	1.2 ± 0.07 ^{B}	1.5 ± 0.43 ^{AB}	1.04 ± 0.10 ^{B}	1.03 ± 0.14 ^{B}	3.1 ± 1.1 ^{A}	0.70 ± 0.18 ^{B}
1-Octanol	3.8 ± 0.64 ^{B}	8.5 ± 0.56 ^{A}	8.8 ± 1.3 ^{A}	8.5 ± 0.61 ^{A}	5.6 ± 0.29 ^{B}	4.5 ± 1.1 ^{B}
**Aldehydes**						
Butanal	1.1 ± 0.11 ^{B}	2.3 ± 0.39 ^{A}	2.2 ± 0.26 ^{A}	1.8 ± 0.11 ^{A}	0.58 ± 0.06 ^{B}	0.46 ± 0.12 ^{B}
Heptanal	2.4 ± 0.19 ^{AB}	3.7 ± 0.60 ^{A}	3.9 ± 0.32 ^{A}	2.6 ± 0.52 ^{AB}	1.9 ± 0.20 ^{B}	1.6 ±0.83 ^{B}
Hexanal	1.7 ± 0.26 ^{BC}	4.2 ± 0.73 ^{AB}	5.5 ± 0.78 ^{A}	3.4 ± 1.1 ^{ABC}	1.2 ± 0.19 ^{C}	1.1 ±0.87 ^{C}
2-Methylbutanal	2.8 ± 0.16 ^{C}	283 ± 6.1 ^{A}	239 ± 17 ^{B}	236 ± 20 ^{B}	1.3 ± 0.07 ^{C}	2.8 ±0.15 ^{C}
3-Methylbutanal	4.2 ± 0.26 ^{D}	128 ± 11 ^{AB}	101 ± 16 ^{B}	150 ± 20 ^{A}	36 ± 3.3 ^{C}	3.6 ±0.52 ^{D}
3-Methyl-2-butenal	3.6 ± 0.07 ^{B}	4.7 ± 0.39 ^{A}	4.9 ± 0.21 ^{A}	4.7 ± 0.04 ^{A}	3.6 ± 0.09 ^{B}	3.2 ±0.54 ^{B}
2-Methylpropanal	5.02 ± 0.21 ^{C}	231 ± 7.8 ^{A}	229 ± 8.3 ^{A}	156 ± 11 ^{B}	5.4 ± 0.25 ^{C}	4.9 ±0.33 ^{C}
Trans-2-Decenal	2.2 ± 0.38 ^{C}	11 ± 2.7 ^{A}	10 ± 4.1 ^{AB}	7.9 ± 1.3 ^{ABC}	3.5 ± 0.18 ^{BC}	3.6 ±2.1 ^{ABC}
**Esters**						
Butyl acetate	0.57 ± 0.08 ^{AB}	2.3 ± 1.01 ^{AB}	3.9 ± 1.9 ^{A}	1.7 ± 1.3 ^{AB}	0.40 ± 0.01 ^{AB}	0.14 ±0.06 ^{B}
Ethyl acetate	nd ^{C}	316 ± 8.4 ^{A}	320 ± 11 ^{A}	296 ± 19 ^{AB}	279 ± 11 ^{B}	nd ^{C}
Ethyl butyrate	0.95 ± 0.07 ^{C}	4.1 ± 0.81 ^{B}	6.3 ± 1.2 ^{A}	2.7 ± 0.48 ^{BC}	1.03 ± 0.13 ^{C}	0.54 ±0.08 ^{C}
Ethyl hexanoate	0.05 ± 0.02	0.05 ± 0.07	0.39 ± 0.30	0.09 ± 0.08	nd	0.04 ±0.01

Ethyl octanoate	nd ^{B}	0.25 ± 0.12 ^{AB}	0.33 ± 0.14 ^{A}	0.17 ± 0.06 ^{AB}	nd ^{B}	0.04 ±0.08 ^{B}
Ethyl formate	nd ^{C}	1.62 ± 0.18 ^{A}	2.04 ± 0.30 ^{A}	1.01 ± 0.14 ^{B}	0.32 ± 0.03 ^{C}	0.28 ±0.08 ^{C}
Ethyl lactate	0.05 ± 0.06 ^{B}	0.50 ± 0.08 ^{AB}	1.01 ± 0.39 ^{A}	0.46 ± 0.07 ^{AB}	0.27 ± 0.02 ^{B}	0.27 ±0.01 ^{B}
Ethyl propionate	nd ^{B}	0.58 ± 0.35 ^{AB}	1.1 ± 0 .70 ^{A}	0.02 ± 0.03 ^{B}	0.12 ± 0.02 ^{AB}	nd ^{B}
Hexyl acetate	0.19 ± 0.02 ^{AB}	0.25 ± 0.09 ^{AB}	0.39 ± 0.12 ^{A}	0.24 ± 0.06 ^{AB}	0.13 ± 0.01 ^{B}	0.13 ± 0.01 ^{B}
2-Methylpropyl acetate	1.8 ± 0.34	0.89 ± 0.68	1.4 ± 0.95	0.46 ± 0.42	0.82 ± 0.23	0.91 ± 0.33
3-Methylbutyl acetate	0.10 ± 0.03 ^{B}	9.3 ± 6.1 ^{A}	17 ± 9.4 ^{A}	5.9 ± 4.8 ^{AB}	0.24 ± 0.02 ^{B}	0.13 ± 0.01 ^{B}
4-Pentenyl acetate	0.15 ± 0.01 ^{B}	2.5 ± 0.27 ^{AB}	5.8 ± 2.8 ^{A}	1.1 ± 0.20 ^{AB}	0.21 ± 0.01 ^{B}	0.19 ± 0.01 ^{B}
Pentyl acetate	0.54 ± 0.06	0.64 ± 0.17	0.78 ± 0.28	0.49 ± 0.20	0.38 ± 0.02	0.28 ± 0.04
2-Phenylethyl acetate	0.04 ± 0.01 ^{AB}	0.27 ± 0.13 ^{AB}	0.47 ± 0.26 ^{A}	0.12 ± 0.14 ^{AB}	0.02 ± 0.02 ^{B}	0.01 ± 0.01 ^{B}
**Ketones**						
2,3-Butanedione	512 ± 41 ^{A}	125 ± 19 ^{C}	130 ± 13 ^{C}	173 ± 43 ^{C}	381 ± 39 ^{B}	392 ± 39 ^{B}
2-Butanone	169 ± 12	191 ± 10	169 ± 22	181 ± 14	154 ± 2.0	168 ± 16
2-Heptanone	53 ± 1.1 ^{B}	57 ± 1.2 ^{A}	57 ± 1.7 ^{A}	55 ± 0.38 ^{AB}	53 ± 0.74 ^{B}	51 ± 5.9
3-Hydroxy-2-butanone	149 ± 26	163 ± 11	161 ± 28	178 ± 13	191 ± 11	182 ± 11
2-Nonanone	6.3 ± 1.1 ^{AB}	13 ± 3.7 ^{A}	11 ± 3.1 ^{A}	7.3 ± 0.44 ^{AB}	3.4 ± 0.47 ^{B}	2.9 ± 0.41 ^{B}
2-Propanone	112 ± 3.5	92 ± 18	76 ± 4.5	92 ± 1.6	83 ± 11	83 ± 13

[1] Relative abundance of each compound was calculated from ratio of the peak area to that of the internal standard (4-methyl-1-pentanol). Different subscripts denote statistically different values ($p < 0.05$) within a raw determined by the one-way ANOVA (Tukey's post-hoc analysis).

Table S2. Concentration of volatile compounds (VOC) in skyr after 14 days incubation of yeasts *Kluyveromyces marxianus* Km1, Km2 and Km3, *Pichia kudriavzevii* Pk1 and *Torulaspora delbrueckii* Td1, and in skyr controls (without added yeasts).

**Compound**	**VOC at Day 14, µg·kg^{-1} [1]**					
	**Control**	***K. marxianus* Km1**	***K. marxianus* Km2**	***K. marxianus* Km3**	***P. kudriavzevii* Pk1**	***T. delbrueckii* Td1**
**Alcohols**						
1-Butanol	38 ± 2.4 ^{A}	24. ± 3.1 ^{B}	23 ± 2.5 ^{B}	24 ± 1.2 ^{B}	18 ± 1.9 ^{B}	21 ± 2.1 ^{B}
2-Butanol	1.4 ± 0.08^{C}	11 ± 3.1 ^{B}	15 ± 0.39 ^{B}	10 ± 1.9 ^{B}	31 ± 1.7 ^{A}	1.3 ± 0.15 ^{C}
1-Hexanol	4.4 ± 0.32 ^{B}	39 ± 1.6 ^{A}	36 ± 2.6 ^{A}	36 ± 0.89 ^{A}	5.9 ± 1.1 ^{B}	4.02 ± 0.15 ^{B}
3-Methyl-1-butanol	17 ± 0.18 ^{C}	1522 ± 53 ^{A}	1669 ± 116 ^{A}	1610 ± 164 ^{A}	374 ± 9.1 ^{B}	32 ± 2.7
3-Methyl-3-buten-1-ol	29 ± 0.61 ^{A}	26 ± 0.44 ^{AB}	24 ± 0.26 ^{B}	26 ± 0.95 ^{AB}	26 ± 0.76 ^{AB}	24 ± 2.1 ^{B}
2-Methyl-1-propanol	0.73 ± 0.08 ^{B}	417 ± 66 ^{A}	482 ± 34 ^{A}	455 ± 45 ^{A}	45 ± 16 ^{B}	1.1 ± 0.10 ^{B}
1-Pentanol	7.3 ± 0.56 ^{B}	20 ± 0.68 ^{A}	19 ± 0.38 ^{A}	20 ± 2.1 ^{A}	9.5 ± 0.73 ^{B}	7.5 ± 0.79 ^{B}
1-Penten-3-ol	0.26 ± 0.02 ^{C}	2.1 ± 0.43 ^{A}	1.6 ± 0.16 ^{AB}	1.9 ± 0.35 ^{A}	1.4 ± 0.14 ^{AB}	0.79 ± 0.28 ^{BC}
2-Phenylethanol	0.18 ± 0.16 ^{B}	1.9 ± 0.88 ^{A}	1.5 ± 0.16 ^{AB}	1.3 ± 0.21 ^{AB}	0.87 ± 0.41 ^{AB}	0.39 ± 0.32 ^{B}
1-Propanol	0.28 ± 0.10 ^{B}	15 ± 6.9 ^{A}	21 ± 2.9 ^{A}	19 ± 8.1 ^{A}	0.92 ± 0.23 ^{B}	0.30 ± 0.06 ^{B}
2-Propanol	0.85 ± 0.48 ^{B}	3.3 ± 2.2 ^{B}	3.3 ± 1.2 ^{B}	2.5 ± 0.34 ^{B}	8.3 ± 0.37 ^{A}	0.58 ± 0.05 ^{B}
1-Octanol	4.5 ± 0.51 ^{B}	18 ± 2.3 ^{A}	15 ± 0.88 ^{A}	14 ± 0.48 ^{A}	6.2 ± 0.71 ^{B}	4.2 ± 0.30 ^{B}
**Aldehydes**						
Butanal	0.44 ± 0.08	0.92 ± 0.41	0.88 ± 0.23	0.81 ± 0.17	0.50 ± 0.13	0.35 ±0.12
Heptanal	1.7 ± 0.14 ^{AB}	1.8 ± 0.28 ^{AB}	1.9 ± 0.31 ^{A}	1.7 ± 0.14 ^{ABC}	1.1 ± 0.23 ^{BC}	0.98 ±0.12 ^{C}
Hexanal	1.1 ± 0.11 ^{AB}	2.6 ± 0.95 ^{AB}	2.9 ± 1.2 ^{A}	2.6 ± 0.45 ^{AB}	1.03 ± 0.34 ^{AB}	0.56 ±0.06 ^{B}
2-Methylbutanal	2.3 ± 0.10 ^{BC}	19 ± 6.1 ^{A}	16 ± 4.6 ^{AB}	28 ± 8.1 ^{A}	0.58 ± 0.41 ^{C}	2.5 ±0.12 ^{BC}
3-Methylbutanal	3.2 ± 0.19 ^{B}	14 ± 1.9 ^{A}	13 ± 2.0 ^{A}	17 ± 3.3 ^{A}	16 ± 1.6 ^{A}	4.04 ±0.24 ^{B}
3-Methyl-2-butenal	2.7 ± 0.09 ^{A}	2.2 ± 0.16 ^{BC}	1.9 ± 0.22 ^{C}	2.0 ± 0.17 ^{C}	2.6 ± 0.08 ^{AB}	2.03 ±0.14 ^{C}
2-Methylpropanal	4.4 ± 0.25 ^{B}	35 ± 15 ^{AB}	32 ± 14 ^{AB}	57 ± 18 ^{A}	5.7 ± 0.47 ^{B}	5.1 ±0.23 ^{B}
Trans-2-Decenal	1.8 ± 0.78 ^{B}	9.3 ± 4.3 ^{A}	6.9 ± 0.54 ^{AB}	6.8 ± 1.5 ^{AB}	2.8 ± 1.1^{B}	3.2 ±1.3 ^{B}
**Esters**						
Butyl acetate	0.57 ± 0.12 ^{B}	1.8 ± 1.5 ^{AB}	4.9 ± 1.5 ^{A}	2.2 ± 2.1 ^{AB}	0.95 ± 0.41 ^{AB}	0.10 ±0.01 ^{B}
Ethyl acetate	nd ^{B}	306 ± 16 ^{A}	317 ± 16 ^{A}	296 ± 3.9 ^{A}	311 ± 12 ^{A}	nd ^{B}
Ethyl butyrate	1.03 ± 0.16 ^{B}	27 ±3.8 ^{A}	38 ± 7.2 ^{A}	26 ± 9.8 ^{A}	1.8 ± 0.45 ^{B}	0.50 ±0.02 ^{B}
Ethyl hexanoate	0.06 ± 0.01 ^{B}	8.2 ± 2.2 ^{AB}	12 ± 5.3 ^{A}	7.6 ± 4.2 ^{AB}	0.01 ± 0.02 ^{B}	0.04 ±0.01 ^{B}

Ethyl octanoate	nd ^{B}	8.5 ± 6.5 ^{A}	21 ± 11 ^{A}	4.5 ± 2.2 ^{A}	nd ^{B}	nd ^{B}
Ethyl formate	0.33 ± 0.02 ^{B}	6.4 ± 3.6 ^{A}	7.9 ± 0.75 ^{A}	6.2 ± 1.1 ^{A}	0.52 ± 0.05 ^{B}	0.34 ±0.01 ^{B}
Ethyl lactate	0.25 ± 0.04 ^{B}	9.4 ± 2.1 ^{A}	9.7 ± 1.2 ^{A}	7.9 ± 2.03 ^{A}	0.56 ± 0.31 ^{B}	0.42 ±0.04 ^{B}
Ethyl propionate	nd ^{B}	1.8 ± 1.1 ^{AB}	6.7 ± 4.9 ^{A}	0.77 ± 0.46 ^{AB}	4.3 ± 2.4 ^{A}	nd ^{B}
Hexyl acetate	0.17 ± 0.01 ^{B}	0.77 ± 0.18 ^{AB}	1.2 ± 0.40 ^{A}	0.76 ± 0.40 ^{AB}	0.20 ± 0.03 ^{B}	0.15 ± 0.01 ^{B}
2-Methylpropyl acetate	2.3 ± 0.18	1.53 ± 1.02	5.5 ± 4.5	3.1 ± 3.4	0.87 ± 0.57	0.73 ± 0.18
3-Methylbutyl acetate	0.05 ± 0.01 ^{B}	18 ± 10 ^{A}	42 ± 23 ^{A}	26 ± 22 ^{A}	2.1 ± 1.1 ^{AB}	0.14 ± 0.01 ^{B}
4-Pentenyl acetate	0.19 ± 0.01 ^{B}	2.3 ± 1.2 ^{AB}	7.2 ± 2.5 ^{A}	1.1 ± 0.03 ^{AB}	0.24 ± 0.05 ^{B}	0.07 ± 0.01 ^{B}
Pentyl acetate	0.60 ± 0.02	0.80 ± 0.24	1.3 ± 0.43	0.79 ± 0.44	0.47 ± 0.14	0.30 ± 0.01
2-Phenylethyl acetate	0.04 ± 0.01 ^{B}	1.2 ± 0.44 ^{AB}	2.7 ± 1.6 ^{A}	1.3 ± 1.1 ^{AB}	0.07 ± 0.05 ^{B}	nd ^{B}
**Ketones**						
2,3-Butanedione	564 ± 14 ^{A}	67 ± 4.1 ^{D}	77 ± 11 ^{D}	71 ± 8.1 ^{D}	128 ± 12 ^{C}	351 ± 28 ^{B}
2-Butanone	117 ± 5.9	86 ± 21	89 ± 31	106 ± 26	67 ± 5.2	121 ± 15
2-Heptanone	49 ± 0.96 ^{B}	53 ± 1.5 ^{AB}	52 ± 0.12 ^{AB}	50 ± 0.53 ^{AB}	51 ± 1.1 ^{AB}	54 ± 2.9 ^{A}
3-Hydroxy-2-butanone	196 ± 11 ^{A}	123 ± 10 ^{BC}	111 ± 18 ^{C}	136 ± 16 ^{BC}	156 ± 10 ^{ABC}	162 ± 11
2-Nonanone	1.26 ± 0.29 ^{C}	21 ± 4.5 ^{A}	22 ± 5.2 ^{A}	17 ± 1.6 ^{AB}	8.6 ± 7.4 ^{ABC}	3.2 ± 1.4 ^{BC}
2-Propanone	84 ± 32	39 ± 19	35 ± 15	44 ± 13	52 ± 5.01	75 ± 3.8

[1] Relative abundance of each compound was calculated from ratio of the peak area to that of the internal standard (4-methyl-1-pentanol). Different subscripts denote statistically different values ($p < 0.05$) within a raw determined by the one-way ANOVA (Tukey's post-hoc analysis).

Table S3. Fermentation and assimilation of lactose, glucose and galactose by yeasts *Kluyveromyces marxianus* Km1, Km2 and Km3, *Pichia kudriavzevii* Pk1 and *Torulaspora delbrueckii* Td1.

**Yeasts**	**Fermentation**[a]			**Assimilation**[b]		
	**Lactose**	**Glucose**	**Galactose**	**Lactose**	**Glucose**	**Galactose**
K. marxianus Km1	+	+	+	+	+	+
K. marxianus Km2	+	+	+	+	+	+
K. marxianus Km3	+	+	+	+	+	+
P. kudriavzevii Pk1	-	+	-	-	+	-
T. delbrueckii Td1	-	+	-	-	+	-

[a] Results are given as + (positive, Durham tube was filled with gas and pH was reduced) and – (negative, no production of air and no changes in pH).

[b] Results are given as + (positive, turbidity was observed as 2+ and 3+ on Wickerhams card), and – (negative, no growth)
